# Cardiovascular risk factors in attention‐deficit/hyperactivity disorder: A family design study of Swedish conscripts

**DOI:** 10.1002/mpr.1930

**Published:** 2022-06-29

**Authors:** Miguel Garcia‐Argibay, Ebba Du Rietz, Catharina A. Hartman, Paul Lichtenstein, Zheng Chang, Cristiano Fava, Samuele Cortese, Henrik Larsson

**Affiliations:** ^1^ School of Medical Science Örebro University Örebro Sweden; ^2^ Department of Medical Epidemiology and Biostatistics Karolinska Institutet Stockholm Sweden; ^3^ Department of Psychiatry University Medical Center Groningen University of Groningen Groningen The Netherlands; ^4^ Department of Medicine University of Verona Verona Italy; ^5^ Department of Clinical Science University of Lund Malmö Sweden; ^6^ School of Psychology University of Southampton Southampton UK; ^7^ Faculty of Medicine Clinical and Experimental Science (CNS and Psychiatry) University of Southampton Southampton Hampshire UK; ^8^ Solent NHS Trust Southampton UK; ^9^ Hassenfeld Children's Hospital at NYU Langone New York University Child Study Center New York City New York USA; ^10^ School of Medicine Division of Psychiatry and Applied Psychology University of Nottingham Nottingham UK

**Keywords:** attention deficit hyperactivity disorder, blood pressure, cardiovascular risk factors, physical fitness, resting heart rate

## Abstract

**Objective:**

(1) investigate the associations of attention‐deficit/hyperactivity disorder (ADHD) with systolic and diastolic blood pressure, resting heart rate, pulse pressure (PP), physical fitness, and BMI; (2) explore whether cardiovascular risk factors and ADHD share genetic and environmental influences; (3) assess if pharmacological treatment for ADHD influences these associations.

**Methods:**

We identified 395,978 individuals born between 1973 and 1991 who had military conscription examinations at a mean age of 18.3 years (SD = 0.57) and their full‐siblings within the same cohort (*N* = 208,060) by linking population‐based registers in Sweden.

**Results:**

Significantly increased risk of ADHD was observed in individuals with low systolic blood pressure (SBP) and PP, low physical fitness, and in those who had overweight or obesity after adjustments (adjusted Odds Ratio [OR] ranging from 1.10 to 1.45). Full siblings of individuals with low SBP, low physical fitness, and obesity were more likely to receive an ADHD diagnosis compared to full siblings without those risk factors (OR ranging from 1.17 to 1.31). Additionally, analyses showed robust associations between ADHD and low SBP, low physical fitness, and obesity, even in ADHD medication‐naïve individuals.

**Conclusions:**

Individuals with several cardiovascular risk factors are more often diagnosed with ADHD, regardless of psychiatric comorbidity. These association are not explained by ADHD pharmacotherapy, rather, they are in part due to shared familial risk factors.

## 1 INTRODUCTION

Attention‐deficit/hyperactivity disorder (ADHD) is a neurodevelopmental condition that has been estimated to affect approximately 3%–7% of the school‐age population (Faraone et al., 2021). There is a growing body of evidence suggesting that impairing symptoms of ADHD persist into adulthood in up to 70% of the cases (Faraone et al., 2021). Attention‐deficit/hyperactivity disorder is characterized by developmentally inappropriate inattention, hyperactivity‐impulsivity, or both, and has a heterogeneous presentation and a multi‐factorial etiology. Psychiatric comorbidity is well recognized in ADHD with high rates of oppositional defiant disorder, conduct disorder, antisocial personality disorder (in adults), substance use disorder (SUD), depressive disorder, and anxiety disorders, amongst others (Faraone et al., 2021; Garcia‐Argibay et al., 2022) leading into a substantial healthcare burden (Garcia‐Argibay et al., 2021).

Individuals with ADHD are at increased risk for cardiovascular disease (CVD) (Spencer et al., 2014). Studies have highlighted a relationship between ADHD and obesity, which increases cardiovascular risk, both in children/adolescents and adults (Cortese et al., 2016). However, associations of ADHD with well‐established cardiovascular risk indicators such as both high systolic blood pressure (SBP) and diastolic blood pressure (DBP), low SBP, elevated resting heart rate (RHR), low physical fitness, and high pulse pressure (PP) (Myers et al., 2002; Tverdal et al., 2008; Yi et al., 2016), remain unclear with contradictory results across studies (Meyer et al., 2017). Research on physical fitness, which is related to cardiovascular function, in ADHD is limited, with only a few studies focusing on fitness levels in individuals with ADHD, suggesting lower physical fitness in adults with ADHD compared to those without ADHD. Prior evidence suggests that reduced physical fitness might be due to low executive function performance and motor skills impairments, as it has been observed in children (Harvey & Reid, 2003), with contradictory results regarding the extent to which ADHD medication dissipates these differences. Nevertheless, other variables may play a role in this association, in particular a sedentary lifestyle and abnormal eating behaviors.

There are different potential explanations for the association between ADHD and indicators for cardiovascular risk that remain to be explored in greater detail. First, psychiatric comorbidity may, at least in part, explain why ADHD is associated with cardiovascular risk factors. Depression, anxiety, and substance abuse are potential negative outcomes of ADHD and known risk factors for cardiometabolic problems. For instance, depression and anxiety (Kemp et al., 2010; Latvala et al., 2016) have been mainly linked with a low heart‐rate variability, but also with abnormalities in blood pressure (Hildru m et al., 2008). Second, familial risk factors may contribute to the association between ADHD and cardiovascular risk factors. Given that ADHD and indicators of cardiovascular risk are highly heritable (Faraone & Larsson, 2019; Polderman et al., 2015), familial risk factors may predispose individuals to both conditions, but this remains to be investigated. Third, the pharmacological treatment for ADHD may influence associations between ADHD and cardiovascular risk factors. Pharmacological ADHD treatment is considered an important component in the management of ADHD. Meta‐analytic evidence showed significantly higher mean SBP and heart rate, but not DBP, in individuals treated with stimulants (Mick et al., 2013), but possible independent associations with ADHD per se have not been addressed in these studies. A better understanding of the link between ADHD and cardiovascular risk factors is critical to appropriately assess and provide adequate treatment for CVD in patients with ADHD.

The main aim of this large‐scale cohort study was to determine the relationship between ADHD in young adults and cardiovascular risk factors as indexed by altered values of SBP, DBP, RHR, PP, body mass index (BMI), and physical fitness. We explored the potential influence of critical covariates, including family socioeconomic status and psychiatric comorbidity (i.e., depression, anxiety, and substance use disorder; SUD). To gain insight on the relationship between ADHD and cardiovascular risk factors, we additionally examined whether ADHD shared familial risk factors with SBP, DBP, RHR, PP, BMI, and physical fitness by assessing the familiar coaggregation. Lastly, in order to assess whether potential associations were due to ADHD medication (rather than the disorder itself), we examined the associations in an unmedicated sample.

## 2 METHODS

### 2.1 Study population

This study was based on the linkage of several Swedish population‐based registers linked by unique personal numbers: The Swedish Military Service Conscription Register (MSCR), the Multi‐Generation Register, Longitudinal Integration Database for Health Insurance and Labor Market Studies (LISA), Prescribed Drug Register (PDR), and National Patient Register (NPR). The MSCR includes extensive medical and psychological assessments for all men at 18 years of age. Military service was obligatory by law to all Swedish men until year 2010 unless individuals were in prison, presented with severe chronic medical or mental conditions or handicaps documented by a medical certificate (approximately 2%–3% annually). All individuals born between 1973 and 1991 who enlisted for military service after 1991 with information on SBP, DBP, RHR, BMI, and physical fitness were identified (*N* = 395,978). Further, using the Multi‐generation Register, we linked these individuals with their biological parents, which allowed us to identify full‐sibling relationships within the same birth cohort (*N* = 208,060) in which the individual and their full sibling shared both of their biological parents. We carried out additional analysis in a unmedicated cohort (before ADHD medication was introduced in Sweden) restricted to military conscription between 1978 and 1991 (*N* = 269,503).

### 2.2 Attention‐deficit/hyperactivity disorder

The presence of ADHD was defined according to the existence of a lifetime diagnosis in the NPR using the International Classification of Diseases versions 9 (1987–1996; ICD‐code 314) or 10 (1997‐present; ICD‐code F90) or a prescription of ADHD medication (methylphenidate: N06BA04, amphetamine: N06BA01, dexamphetamine: N06BA02, atomoxetine: N06BA09, lisdexamfetamine: N06BA12) from the PDR. The NPR provides diagnoses for psychiatric inpatient care since 1973 and outpatient care since 2001 up until 2013, and the PDR includes drug prescriptions dispensations from 2005 to 2013.

### 2.3 Cardiovascular risk factors

Blood pressure (BP) and RHR were measured while in a supine position after 10 min of rest during the first day of the conscription examination. Data points were regarded as outliers and potential data errors, and as such excluded from the analysis, if SBP was below 45 and above 200 mm Hg, DBP was below 40 and above 120 mm Hg, and RHR was below 35 and above 145 beats per minute (bpm). Pulse pressure was calculated as the difference between SBP and DBP. Variables were categorized into low, medium, and high based on the 25^th^ and 75^th^ percentiles (first, second and third, and fourth quartile, respectively), and the medium class was used as the reference group.

Body mass index was calculated using weight and height measurements (kg/m^2^) from the military conscription register values and categorized according to WHO criteria into low weight (<18.5), normal weight (18.5 ≤ BMI <25; used as reference group), overweight (25 ≤ BMI <30), and obesity (BMI ≥30) (World Health Organization, 2000). Physical fitness was assessed using the cycle ergonometric test in which heart rate was measured during 5 min of submaximal exercise and then divided by body mass (kg) to take into account body size. Physical fitness was categorized into low, medium, and high based on the 25^th^ and 75^th^ percentiles and the medium class was used as the reference group.

### 2.4 Covariates

Birth year and sex were obtained from the Total Population Register. Household socioeconomic status was defined as whether any parent of a child had college education or not (binary variable yes/no) using the database LISA. These were all included as covariates in the analysis. Lifetime diagnoses of substance use disorder (SUD; ICD‐10: F10‐F19; ICD‐9: 303–305; ICD‐8: 304), depressive disorders (ICD‐10: F32–34; ICD‐9: 296B, 300E; ICD‐8: 296.2, 296.9, 298.0, 300.4), and anxiety disorders (ICD‐10: F40‐F41; ICD‐9: 300A, 300C, ICD‐8: 300.0) were obtained from the NPR and were dichotomized into binary variables.

### 2.5 Statistical analysis

In order to determine the relationship between ADHD and our set of risk factors (i.e., SBP, DBP, RHR, BMI, physical fitness, and PP), a series of logistic regression models were performed. Results were displayed as odds ratios (ORs) together with their 95% confidence intervals (CI). The statistical significance was set at *p* < 0.05. Data preparation and analyses were performed using SAS software version 9.4 (SAS Institute Inc., Cary, NC) and R 4.0.2, respectively.

### 2.6 Within‐individual association

The odds of having an ADHD diagnosis in individuals with our sets of predictors were estimated. In the first model, estimates were adjusted for birth year to adjust for potential cohort effects and differences in follow‐up length. In the second model, further adjustment was made for sex, family socioeconomic status, depression, anxiety, and SUD.

### 2.7 Between‐sibling associations

In order to investigate the presence of a shared familial component between ADHD and cardiovascular risk factors, we estimated the ORs of having ADHD in individuals whose full sibling had low or high values of our predictors compared to the reference group (normal values for BMI and medium values for SBP, DBP, RHR, physical fitness, and PP). Higher risk of ADHD in siblings of individuals with high or low predictor values indicates that familial (i.e., genetic and environmental) factors shared among siblings contribute to the co‐occurrence of conditions. Estimates were adjusted for birth year. In order to account for the non‐independence of family‐clustered observations, the Huber‐White cluster‐robust sandwich estimator was used allowing for intragroup correlation for the standard errors (SE).

### 2.8 Associations before the introduction of ADHD medication

Due to the potential effect of ADHD medication on BP and heart rate (Mick et al., 2013), we conducted additional analyses restricted to military conscription (*N* = 269,503) between 1978 and 1991, that is, before ADHD medication was introduced in Sweden, to examine whether associations were influenced by ADHD medication. Estimates were first adjusted for birth year and then, in a second model, further adjusted for sex, family socioeconomic status, depression, anxiety, and SUD.

### 2.9 Sensitivity analysis

In order to assess the robustness of our findings, several sensitivity analyses were carried out. First, BMI and RHR were included separately as covariates to control for the effect of BMI on associations between ADHD and SBP, DBP, RHR, PP, and physical fitness, and RHR was included to adjust for the potential white‐coat effect on SBP and DBP. Second, due to the high level of missing data for physical fitness (37.5%) in the main sample (i.e., military conscription after 1992), within‐individual analyses were performed after missing values were imputed. K‐nearest neighbor (kNN) imputation was carried out using 5 neighbors based on the Gower's distance function. Third, analyses were restricted to military conscriptions recruited after year 2000 to include individuals with in and outpatient data. Lastly, diagnoses were defined as having a diagnosis before military conscription and before age 25 (compared with lifetime in the main analysis).

## 3 RESULTS

### 3.1 Description of the study population

Among 395,978 index individuals included in the study, the vast majority were men (97.3%) and the mean age at military conscription was 18.31 years (SD = 0.57). The estimated lifetime prevalence of ADHD was 1.3% for the index individuals and 5.2% for full siblings of individuals with ADHD, with a mean age at diagnosis of 27.7 years (SD = 5.88). The mean age difference at conscription between the index individuals and their siblings was 0.03 months (SD = 8.68). Additional descriptive information can be observed in Table 1 and Table S1 for the main and unmedicated cohorts, respectively.

**TABLE 1:** Descriptive statistics of the study population

	Level	Overall	Stratified by ADHD		
			Without ADHD	With ADHD	*p*
N		395,978	390,836	5142	
Demographics					
Gender (%)	Male	385,306 (97.3)	380,290 (97.3)	5016 (97.5)	0.295
	Female	10,672 (2.7)	10,546 (2.7)	126 (2.5)	
Age at conscription, years		18.31 (0.57)	18.31 (0.57)	18.38 (0.65)	<0.001
Age at diagnosis of ADHD		27.7 (5.88)	‐	27.7 (5.88)	
Height, cm		179.52 (6.91)	179.53 (6.91)	178.80 (7.00)	<0.001
BMI (%)	Normal	312,572 (78.9)	308,736 (79.0)	3836 (74.6)	<0.001
	Low	11,151 (2.8)	10,961 (2.8)	190 (3.7)	
	Overweight	58,667 (14.8)	57,825 (14.8)	842 (16.4)	
	Obese	13,588 (3.4)	13,314 (3.4)	274 (5.3)	
Family education (%)	Without college	294,438 (74.4)	289,815 (74.2)	4623 (89.9)	<0.001
	With college	101,540 (25.6)	101,021 (25.8)	519 (10.1)	
Psychiatric disorders					
Depression (%)	No	382,434 (96.6)	379,294 (97.0)	3140 (61.1)	<0.001
	Yes	13,544 (3.4)	11,542 (3.0)	2002 (38.9)	
Anxiety (%)	No	382,942 (96.7)	379,797 (97.2)	3145 (61.2)	<0.001
	Yes	13,036 (3.3)	11,039 (2.8)	1997 (38.8)	
SUD (%)	No	378,786 (95.7)	375,577 (96.1)	3209 (62.4)	<0.001
	Yes	17,192 (4.3)	15,259 (3.9)	1933 (37.6)	
Cardiovascular risk factors					
SBP, mm Hg (%)	Normal (120–136)	221,259 (55.9)	218,425 (55.9)	2834 (55.1)	
	Low (<120)	65,266 (16.5)	64,170 (16.4)	1096 (21.3)	
	High (>136)	109,453 (27.6)	108,241 (27.7)	1212 (23.6)	
DBP, mm Hg (%)	Normal (62–75)	213,439 (53.9)	210,747 (53.9)	2692 (52.4)	
	Low (<62)	99,617 (25.2)	98,312 (25.2)	1305 (25.4)	
	High (>75)	82,922 (20.9)	81,777 (20.9)	1145 (22.3)	
RHR, bpm (%)	Normal (64–81)	202,084 (51.0)	199,445 (51.0)	2639 (51.3)	
	Low (<64)	110,877 (28.0)	109,462 (28.0)	1415 (27.5)	
	High (>81)	83,017 (21.0)	81,929 (21.0)	1088 (21.2)	
PP (%)	Normal (53–68)	198,486 (50.1)	195,926 (50.1)	2560 (49.8)	
	Low (<53)	101,834 (25.7)	100,250 (25.7)	1584 (30.8)	
	High (>68)	95,658 (24.2)	94,660 (24.2)	998 (19.4)	
Physical fitness (%)	Normal (3.75–4.64)	123,509 (31.2)	122,226 (31.3)	1283 (25.0)	
	Low (<3.74)	62,091 (15.7)	61,136 (15.6)	955 (18.6)	
	High (>4.65)	61,828 (15.6)	61,384 (15.7)	444 (8.6)	
	Missing	148,550 (37.5)	146,090 (37.4)	2460 (47.8)	
Imputed physical fitness (%)	Normal	198,509 (50.1)	195,835 (50.1)	2674 (52.1)	
	Low	93,694 (23.7)	92,149 (23.6)	1545 (30.1)	
	High	103,608 (26.2)	102,695 (26.3)	913 (17.8)	

*Note*: Table values are mean (standard deviation) and percent for continuous and categorical variables, respectively. Chi‐square tests were used for categorical outcomes.

Abbreviations: ADHD, attention‐deficit/hyperactivity disorder; BMI, body mass index; DBP, diastolic blood pressure; PP = pulse pressure; RHR = resting heart rate; SUD = substance use disorder; bpm = beats per minute; SBP = systolic blood pressure.

### 3.2 Within‐individual associations

Among index individuals, ADHD was positively associated with low SBP (OR = 1.28, 95% CI [1.21–1.35]), high DBP (OR = 1.09, 95% CI [1.02–1.16]), low PP (OR = 1.19, 95% CI [1.13–1.26]), low physical fitness (OR = 1.80, 95% CI [1.65–1.95]), being overweight (OR = 1.20, 95% CI [1.13–1.28]), and obese (OR = 1.64, 95% CI [1.52–1.77]), while adjusting for birth year (Table 2). Conversely, ADHD was negatively associated with high SBP (OR = 0.89, 95% CI [0.82–0.96]), high PP (OR = 0.82, 95% CI [0.75–0.89]), and high physical fitness (OR = 0.53, 95% CI [0.45–0.61]), whereas no significant association was found between ADHD and RHR or low DBP. After further adjustment for sex, highest education of either parent, depression, anxiety, and SUD, the associations remained significant for all associations except for high DBP (Table 2).

**TABLE 2:** Association between systolic blood pressure (SBP), diastolic blood pressure (DBP), resting heart rate (RHR), pulse pressure (PP), physical fitness, body mass index (BMI) and ADHD

Variable		Within‐individual associations with ADHD		Cross‐sibling associations with ADHD	
		Birth‐year adjusted	Adjusted OR^a^	Crude	Birth‐year adjusted
SBP	Low	**1.28** (**1.21–1.35)**	**1.20** (**1.12–1.27)**	**1.17** (**1.05–1.30)**	**1.17** (**1.05–1.30)**
	High	**0.90** (**0.83–0.96)**	**0.93** (**0.85‐0.99)**	**0.89** (**0.80‐0.98)**	**0.89** (**0.80–0.98)**
DBP	Low	1.01 (0.94–1.08)	1.04 (0.97–1.11)	1.01 (0.90–1.10)	1.01 (0.90–1.10)
	High	**1.09** (**1.03–1.17)**	1.05 (0.97–1.12)	1.08 (0.98–1.20)	1.08 (0.98–1.20)
RHR	Low	1.01 (0.95–1.08)	1.04 (0.97–1.11)	0.95 (0.86–1.04)	0.95 (0.86–1.04)
	High	0.98 (0.91–1.05)	0.95 (0.87–1.02)	0.94 (0.84–1.05)	0.94 (0.84–1.05)
PP	Low	**1.19** (**1.13–1.26)**	**1.11** (**1.04–1.18)**	1.09 (0.99–1.19)	1.09 (0.99–1.19)
	High	**0.82** (**0.75–0.89)**	**0.89** (**0.81–0.97)**	**0.84** (**0.76–0.93)**	**0.84** (**0.76–0.93)**
Physical fitness	Low	**1.58** (**1.49–1.66)**	**1.31** (**1.22–1.40)**	**1.24** (**1.09–1.40)**	**1.24** (**1.09–1.40)**
	High	**0.62** (**0.51–0.72)**	**0.81** (**0.69–0.92)**	**0.83** (**0.72–0.95)**	**0.82** (**0.72–0.94)**
BMI	Low	**1.31** (**1.17–1.46)**	1.02 (0.86–1.18)	1.14 (0.90–1.45)	1.14 (0.90–1.45)
	Overweight	**1.20** (**1.13–1.28)**	**1.17** (**1.09–1.25)**	1.11 (0.99–1.25)	1.11 (0.99–1.25)
	Obese	**1.65** (**1.53–1.78)**	**1.45** (**1.32–1.59)**	**1.31** (**1.07–1.62)**	**1.31** (**1.07–1.62)**

*Note*: Crude and adjusted associations within‐individuals and cross‐siblings.

*Note*: ^a^Model adjusted for birth year, sex, highest education of either parent, depression, anxiety, and substance use disorder. Crude: Model not adjusted for covariates. Reference class was set to normative/median values. Bolded estimates are significant at α < 0.05.

Abbreviations: ADHD, attention‐deficit/hyperactivity disorder; DBP, diastolic blood pressure; OR, Odds Ratio.

### 3.3 Between‐sibling associations

Associations between full siblings displayed an increased risk of having ADHD in full siblings of individuals with low SBP (OR = 1.17, 95% CI [1.05–1.30]), low physical fitness (OR = 1.24, 95% CI [1.09–1.40]), and obesity (OR = 1.31, 95% CI [1.07–1.62]) compared with individuals in the medium group. Conversely, a lower risk was observed for full siblings of cases with high SBP (OR = 0.89, 95% CI [0.80–0.98]), high PP (OR = 0.84, 95% CI [0.76–0.93]), and high physical fitness (OR = 0.82, 95% CI [0.72–0.94]). The between‐siblings associations of ADHD with measures of RHR and DBP were not significant. Table 2 displays additional estimates.

### 3.4 Associations before the introduction of ADHD medication

In order to examine whether associations could be influenced by ADHD medication, we compared the estimates with military conscriptions before (*N* = 269,503) and after ADHD medication was introduced in 1992 (in main analyses; *N* = 395,978). Results displayed robust patterns of associations before and after 1992, and associations remained significant before 1992 for high (negative association) and low SBP, PP, low physical fitness, and obesity with ADHD (Table 3). Results revealed a significant association between ADHD and low RHR only for measures obtained before 1992.

**TABLE 3:** Association between systolic blood pressure (SBP), diastolic blood pressure (DBP), resting heart rate (RHR), pulse pressure (PP), physical fitness, body mass index (BMI) and ADHD

Variable		Within‐individual associations with ADHD	
		Birth‐year adjusted	Adjusted OR^a^
SBP	Low	**1.23** (**1.13–1.33)**	**1.14** (**1.03–1.25)**
	High	**0.82** (**0.71–0.93)**	**0.83** (**0.72–0.94)**
DBP	Low	0.93 (0.84–1.02)	0.98 (0.89–1.08)
	High	1.02 (0.91–1.14)	1.00 (0.88–1.12)
RHR	Low	0.99 (0.90–1.09)	1.04 (0.94–1.14)
	High	**0.83** (**0.72–0.94)**	**0.77** (**0.66–0.89)**
PP	Low	**1.13** (**1.04–1.23)**	1.09 (0.99–1.19)
	High	**0.79** (**0.69–0.89)**	**0.86** (**0.75–0.96)**
Physical fitness	Low	**1.35** (**1.27–1.44)**	**1.13** (**1.04–1.22)**
	High	**0.71** (**0.50–0.92)**	0.83 (0.61–1.05)
BMI	Low	**1.24** (**1.07–1.42)**	0.99 (0.81–1.17)
	Overweight	0.95 (0.80–1.10)	0.89 (0.73–1.05)
	Obese	**1.53** (**1.25–1.80)**	**1.45** (**1.16–1.73)**

*Note*: Crude and adjusted associations for conscription measures obtained before 1992 (*N* = 208,060).

*Note*: ^a^Model adjusted for birth year, sex, highest education of either parent, depression, anxiety, and SUD. Reference class was set to normative/median values. Bolded estimates are significant at α < 0.05.

Abbreviations: ADHD, attention‐deficit/hyperactivity disorder; OR, Odds Ratio; SUD, substance use disorder.

### 3.5 Sensitivity analysis

ORs and CIs remained nearly unchanged after further adjusting for BMI and RHR (Table S2 displays estimates after adjustment for BMI and RHR of the index individual and full sibling). Estimates after kNN imputation for physical fitness remained similar (overlapping CIs compared with the original estimates) before (low: *b* = 1.11, 95% CI [1.01, 1.21], high: *b* = 0.81, 95% CI [0.62, 0.98]) and after medication was introduced (low: *b* = 1.21, 95% CI [1.14, 1.28], high: *b* = 0.87, 95% CI [0.79, 0.95]). Finally, estimates remained unchanged regardless of diagnoses definition (i.e., at conscription, before age 25, or lifetime; Table S3) or conscriptions after year 2000, with the exception of PP, which was no longer significant when using diagnoses at conscription or before age 25.

## 4 DISCUSSION

In this population‐based cohort study, we observed a significant, positive association between low SBP, low PP, low physical fitness, overweight/obesity and ADHD after adjusting for comorbid psychiatric conditions (i.e., depression, anxiety, and SUD) and family education. Moreover, we found that full siblings of individuals with low SBP, low physical fitness, and who had obesity were also more likely to be diagnosed with ADHD compared to full siblings of individuals in the reference group. Additional analyses showed that individuals with low levels of SBP, PP, physical fitness, and obese were more likely to be diagnosed with ADHD even before ADHD medication was introduced in Sweden, which highlights that ADHD per se, rather than its pharmacologic treatment, is an important risk factor for several important indicators of cardiovascular risk. The finding that individuals with ADHD, on average, present with lower SBP is novel, with only one prior study reporting similar results (Meyer et al., 2017). Our results, whilst confirming this association, point to the need of additional replications.

Our results are in line with prior research on ADHD and obesity (Cortese et al., 2016), wherein an increased risk for ADHD in individuals who are overweight or obese was reported. In addition, we observed that individuals with ADHD also displayed lower physical fitness even after adjusting for BMI. Given that physical hyperactivity is one of the core symptoms of ADHD, it would be reasonable to think that those with ADHD are unlikely to be overweight or present with low physical fitness. However, evidence shows that individuals with ADHD are more likely to live a sedentary lifestyle and engage in other unhealthy behavior such as smoking or disordered eating (Quesada et al., 2018). These findings are clinically important as previous studies found that fitness capacity and obesity are predictors of mortality and impaired cardiac function (Myers et al., 2002). Importantly, we showed that the positive relationship between obesity and low physical fitness with ADHD was present before ADHD medication was introduced, indicating that ADHD medication does not explain the observed associations of ADHD with BMI and physical fitness. Furthermore, siblings of index individuals with low physical fitness, and who were obese, were at higher risk for ADHD compared with siblings of index individuals in the reference group. This familial association suggests that there may be shared genetic and/or environmental factors that influence both ADHD and the cardiovascular risk factors. One explanation could be that neurobiological substrates are implicated across conditions of ADHD, overweight/obesity, and low physical fitness. These substrates have also been observed in other comorbid disorders such as anxiety, depression, and SUD (Farb & Ratner, 2014; Katzman & Sternat, 2016).

A growing body of evidence suggests that numerous neurotransmitters and brain structures are involved in ADHD (Cortese, 2012), including a monoaminergic hypofunction involving dopamine and norepinephrine pathways. A dysfunction in these neurotransmitters affects different brain structures that are linked to blood pressure as neuroendocrine regulation of blood pressure requires integrated actions of multitude brain structures such as cortical regions, hypothalamus, locus coeruleus, prefrontal cortex, and basal ganglia (Buijs, 2013; Kollins & Adcock, 2014). This dysregulation has also been observed in other psychiatric disorders such as depression and anxiety disorders (Hildrum et al., 2008) and recent research have shown a significant link between severe psychiatric conditions (i.e., schizophrenia, bipolar disorder, and depression) and CVD (Correll et al., 2017), however, less is known about CVD in ADHD. Our results are in line with this notion, suggesting that individuals with ADHD might experience blood pressure dysregulation, specifically lower SBP. High SBP is a well‐established risk factor for CVD and mortality, however, although research on low SBP is scarce, observational studies showed that low SBP increases mortality in adolescents, adults, and the elderly (Sundstrom et al., 2011; Yi et al., 2016). Similarly, both low and high PP has been linked to an increase in mortality in patients with heart failure (Yildiran et al., 2010). Our results could reflect a decreased cardiac function resulting in a decrease in stroke volume and ultimately a decrease in SBP.

Lastly, the present study did not find support for the association of ADHD with DBP and RHR, although previous research has reported a small increase in SBP (+2.0 mmHg) and RHR (+5.7 bpm) associated with the use of ADHD medication (Mick et al., 2013). Further research is required to develop a deeper understanding of the relationships between ADHD and cardiovascular risk factors, and whether the increased risk is present before the initiation of ADHD medication and if there is an additive effect. Future studies should replicate the observed association between ADHD and lower SBP and attempt to unravel the mechanisms that explain the association.

This study presents several strengths including a large sample size (∼600,000 participants), a population‐based design, and the use of objective and detailed measures of cardiovascular risk factors. Moreover, the study population largely represents the male population given that only a very small amount did not qualify for military conscription (2%–3% annually). However, the study should be interpreted in light of its limitations. Given our definition of ADHD using diagnoses and medication prescriptions and the low prevalence ratio in our sample (1.3%) compared to the reported in the literature (Faraone et al., 2021), our results may mainly generalize to individuals with more severe forms of ADHD. The low prevalence is explained by the use of ICD‐10 hyperkinetic disorder, which is roughly equivalent to the combined presentation of ADHD as per the DSM‐5, the fact that the older individuals in the cohort did not get a diagnosis given that the disorder was not diagnosed several decades ago, and that many individuals with ADHD are not in the care facilities, if any, covered by the registers. In addition, ADHD was not generally diagnosed in Sweden before the 1990s and therefore, some of the ADHD cases in our sample might had been misclassified as not having ADHD. This would have potentially underestimated the reported associations and decreased the statistical power in the sensitivity analysis before 1992. Moreover, given that only males were obliged to enter the military (up to 2010), the generalizability of these findings to females is limited due to the small proportion of females in our sample (2.7%). Future research should address whether these results can be extrapolated to females or whether they are restricted to males. Sex differences are of special importance due to an under‐identification of females diagnosed with ADHD caused by referral bias, and the possibility that clinical samples might mask gender differences as only females with significant impairments are referred to healthcare. Furthermore, it was not possible to assess to what extent different ADHD subtypes display the same obtained pattern of results. Lastly, it is worth noting that the magnitude of the associations was overall small (range of aOR = 0.81–1.45) and future research should assess whether these associations translate into clinically relevant adverse outcomes. Although several sensitivity analyses showed robust associations, we cannot rule out the possibility that the association between low SBP and ADHD was influenced by a loss of individuals (due to death prior to conscription) with high SBP values and in turn affecting the percentiles. The large sample size and the young age of the sample indicate that this type of bias probably only had a minimal impact on the observed associations.

## 5 CONCLUSIONS

The results of this research revealed that individuals with ADHD present a potential increased risk for several cardiovascular risk factors, such as being overweight/obese and having lower physical fitness levels, which highlights the importance of early and frequent monitoring of SBP, physical fitness, and BMI in patients with ADHD. Moreover, our study revealed that individuals with ADHD may present lower levels of SBP and PP compared to the general population, which might reflect blood pressure dysregulation. Clinicians should therefore be aware that certain pre‐existing conditions might be present before starting ADHD medication, and thus, assessing cardiovascular risk factors independently from medication is recommended. Lastly, ADHD and SBP, PP, physical fitness, and obesity share familial risk factors suggesting that the association between ADHD and the aforementioned cardiovascular risk factors may be explained by common pathophysiologic mechanisms, including genetic background and joint pathways. Future research aiming to elucidate shared genetic and environmental risk factors and pleiotropic genetic variants for ADHD and cardiovascular risk factors is therefore needed.

## CONFLICT OF INTEREST

Henrik Larsson reported receiving grants from Shire/Takeda Pharmaceuticals during the conduct of the study; personal fees from and serving as a speaker for Shire/Takeda Pharmaceuticals and Evolan Pharma AB outside the submitted work; and sponsorship for a conference on attention‐deficit/hyperactivity disorder from Shire Pharmaceuticals outside the submitted work. Ebba Du Rietz has served as a consultant for Shire Sweden AB (fully owned subsidiary of Takeda Pharmaceutical Company Limited) outside the submitted work. Samule Cortese declares honoraria and reimbursement for travel and accommodation expenses for lectures from the following non‐profit associations: Association for Child and Adolescent Central Health (ACAMH), Canadian ADHD Alliance Resource (CADDRA), British Association of Pharmacology (BAP), and from Healthcare Convention for educational activity on ADHD. The remaining authors declare having no conflict of interest.

## AUTHOR CONTRIBUTIONS

Miguel Garcia‐Argibay and Henrik Larsson conceptualized and designed the study. Miguel Garcia‐Argibay conducted literature search, analyzed the data, and drafted the manuscript. Henrik Larsson and Ebba Du Rietz provided supervision. All authors contributed to the interpretation of results, reviewing, and editing of the final manuscript, and had responsibility in deciding to submit the manuscript for publication. Miguel Garcia‐Argibay attests that all listed authors meet authorship criteria and that no other individuals meeting the criteria have been omitted.

## ETHICS STATEMENTS

The study had ethical approval from the Regional Ethical Review Board in Stockholm, Sweden (Dnr 2013/862–31/5). Requirement for informed consent was waived for the current study because it was a secondary analysis of existing data. The investigation conforms to the 1964 Helsinki declaration and its later amendments or comparable ethical standards.

## Supporting information

Supporting Information S1

## Data Availability

The Public Access to Information and Secrecy Act in Sweden prohibits us from making individual level data publicly available. Researchers who are interested in replicating our work can apply for individual level data at Statistics Sweden: www.scb.se/en/services/guidance‐for‐researchers‐and‐universities/.
