# Supplementary material for: Cardiovascular risk factors in attention‐deficit/hyperactivity disorder: A family design study of Swedish conscripts
Source: Int J Methods Psychiatr Res. 2022 Jun 29;31(4):e1930. doi: 10.1002/mpr.1930 (PMC9720218; doi:10.1002/mpr.1930)
Supplement: Supplementary file 1 — Supporting Information S1 [file MPR-31-e1930-s001.docx]

**Supplementary materials**

Cardiovascular risk factors in attention-deficit/hyperactivity disorder: a family design study of Swedish conscripts. Garcia-Argibay, M., Du Rietz, E., Lichtenstein, P., Hartman, C., Chang, Z., Fava, C., Cortese, S., Larsson, H.

**Table 1.** Descriptive statistics of the study population before 1992

|  | Level | Overall | Stratified by ADHD | |  |
| --- | --- | --- | --- | --- | --- |
|  |  |  | Without ADHD | With ADHD | *p* |
| N |  | 269 503 | 267 148 | 2 355 |  |
| *Demographics* |  |  |  |  |  |
| Gender (%) | Male | 269 069 (99.8) | 266 719 (99.8) | 2 350 (99.8) | 0.715 |
|  | Female | 434 (0.2) | 429 (0.2) | 5 (0.2) |  |
| Age at conscription, years |  | 18.14 (0.50) | 18.14 (0.50) | 18.18 (0.59) | <0.001 |
| Age at diagnosis of ADHD |  | 46 (3.27) | - | 46 (3.27) |  |
| Height, cm |  | 179.22 (6.53) | 179.23 (6.53) | 178.37 (6.60) | <0.001 |
| BMI (%) | Normal | 229 884 (85.3) | 227 904 (85.3) | 1 980 (84.1) | 0.001 |
|  | Low | 12 859 (4.8) | 12 722 (4.8) | 137 (5.8) |  |
|  | Overweight | 22 710 (8.4) | 22 525 (8.4) | 185 (7.9) |  |
|  | Obese | 4 050 (1.5) | 3 997 (1.5) | 53 (2.3) |  |
| Family education (%) | Without college | 190 157 (70.6) | 188 172 (70.4) | 1 985 (84.3) | <0.001 |
|  | With college | 79 346 (29.4) | 78 976 (29.6) | 370 (15.7) |  |
| *Psychiatric disorders* |  |  |  |  |  |
| Depression (%) | No | 258 179 (95.8) | 256 842 (96.1) | 1 337 (56.8) | <0.001 |
|  | Yes | 11 324 (4.2) | 10 306 (3.9) | 1 018 (43.2) |  |
| Anxiety (%) | No | 259 595 (96.3) | 258 154 (96.6) | 1 441 (61.2) | <0.001 |
|  | Yes | 9 908 (3.7) | 8 994 (3.4) | 914 (38.8) |  |
| SUD (%) | No | 253 695 (94.1) | 252 530 (94.5) | 1 165 (49.5) | <0.001 |
|  | Yes | 15 808 (5.9) | 14 618 (5.5) | 1 190 (50.5) |  |
| *Cardiovascular risk factors* |  |  |  |  |  |
| SBP, mm Hg (%) | Normal | 160 477 (59.5) | 159 078 (59.5) | 1 399 (59.4) |  |
|  | Low | 49 581 (18.4) | 49 051 (18.4) | 530 (22.5) |  |
|  | High | 59 445 (22.1) | 59 019 (22.1) | 426 (18.1) |  |
| DBP, mm Hg (%) | Normal | 124 969 (46.4) | 123 854 (46.4) | 1 115 (47.3) |  |
|  | Low | 98 760 (36.6) | 97 936 (36.7) | 824 (35.0) |  |
|  | High | 45 774 (17.0) | 45 358 (17.0) | 416 (17.7) |  |
| RHR, bpm (%) | Normal | 141 128 (52.4) | 139 846 (52.3) | 1 282 (54.4) |  |
|  | Low | 71 132 (26.4) | 70 489 (26.4) | 643 (27.3) |  |
|  | High | 57 243 (21.2) | 56 813 (21.3) | 430 (18.3) |  |
| PP (%) | Normal | 116 210 (43.1) | 115 156 (43.1) | 1 054 (44.8) |  |
|  | Low | 65 409 (24.3) | 64 739 (24.2) | 670 (28.5) |  |
|  | High | 87 884 (32.6) | 87 253 (32.7) | 631 (26.8) |  |
| Physical fitness (%) | Normal | 110 666 (41.1) | 109 830 (41.1) | 836 (35.5) |  |
|  | Low | 141 329 (52.4) | 139 908 (52.4) | 1 421 (60.3) |  |
|  | High | 17 435 (6.5) | 17 339 (6.5) | 96 (4.1) |  |
|  | Missing | 73 (0.0) | 71 (0.0) | 2 (0.1) |  |

Note. Table values are mean (standard deviation) and percent for continuous and categorical variables, respectively. Chi-square tests were used for categorical outcomes. BMI = body mass index; RHR = resting heart rate; SUD = substance use disorder; bpm = beats per minute; SBP = systolic blood pressure; DBP = diastolic blood pressure; PP = pulse pressure.

**Table 2**. Association between systolic blood pressure (SBP), diastolic blood pressure (DBP), resting heart rate (RHR), pulse pressure (PP), physical fitness, body mass index (BMI) and ADHD. Adjusted OR (95% CI) of the individual and full sibling.

| Variable |  | Within-individual associations with ADHD | | Cross-sibling associations with ADHD | |
| --- | --- | --- | --- | --- | --- |
|  |  | BMI-adjusted | RHR-adjusted | BMI-adjusted | RHR-adjusted |
| SBP | Low | **1.22 (1.14-1.29)** | **1.19 (1.12-1.27)** | **1.18 (1.06-1.31)** | **1.17 (1.05-1.31)** |
|  | High | **0.91 (0.83-0.98)** | 0.93 (0.86-1.00 | **0.88 (0.79-0.97)** | **0.89 (0.80-0.98)** |
| DBP | Low | 1.04 (0.97-1.11) | 1.03 (0.96-1.11) | 1.00 (0.91-1.10) | 1.00 (0.90-1.10) |
|  | High | 1.03 (0.96-1.11) | 1.05 (0.98-1.13) | 1.08 (0.97-1.20) | 1.09 (0.98-1.20) |
| RHR | Low | **1.05 (0.98-1.12)** | - | **0.95 (0.86-1.05)** | **-** |
|  | High | **0.93 (0.85-1.01)** | - | **0.93 (0.84-1.04)** | **-** |
| PP | Low | **1.12 (1.05-1.19)** | **1.10 (1.03-1.17)** | 1.09 (0.99-1.20) | 1.09 (0.99-1.20) |
|  | High | **0.88 (0.80-0.96)** | **0.89 (0.81-0.97)** | **0.84 (0.75-0.93)** | **0.84 (0.75-0.93)** |
| Physical fitness | Low | **1.28 (1.19-1.38)** | **1.33 (1.24-1.42)** | **1.26 (1.10-1.45)** | **1.25 (1.10-1.42)** |
|  | High | **0.81 (0.70-0.93)** | **0.80 (0.69-0.92)** | **0.82 (0.71-0.94)** | **0.82 (0.71-0.94)** |

*Note.* Cross-sibling associations adjusted for birth year; Within-individual associations adjusted for birth year, sex, highest education of either parent, depression, anxiety, and SUD. Reference class was set to normative/median values. Bolded estimates are significant at α < .05.

**Table 3.** Association between systolic blood pressure (SBP), diastolic blood pressure (DBP), resting heart rate (RHR), pulse pressure (PP), physical fitness, body mass index (BMI) and ADHD using different diagnoses definitions. Adjusted OR (95% CI).

|  | Conscription after 2000 | | Diagnoses up to age 18 | | Diagnoses up to age 25 | |
| --- | --- | --- | --- | --- | --- | --- |
| Variable |  | After 2000 | After 1992 | Before 1992 | After 1992 | Before 1992 |
| SBP | Low | **1.16 (1.02-1.31)** | **1.53 (1.28-1.79)** | **1.21 (1.11-1.32)** | **1.28 (1.13-1.44)** | **1.20 (1.10-1.30)** |
| SBP | High | 1.01 (0.89-1.12) | 0.95 (0.73-1.18) | 0.84 (0.73-0.95) | 1.03 (0.9-1.16) | 0.84 (0.73-0.95) |
| DBP | Low | 0.99 (0.87-1.13) | 1.05 (0.81-1.29) | 0.93 (0.84-1.02) | 1.10 (0.96-1.24) | 0.94 (0.84-1.03) |
| DBP | High | 1.11 (0.99-1.24) | 1.22 (0.98-1.46) | 1.02 (0.91-1.13) | **1.25 (1.11-1.39)** | 1.02 (0.9-1.13) |
| RHR | Low | 0.96 (0.84-1.08) | 1.08 (0.86-1.30) | 0.99 (0.91-1.10) | 0.92 (0.79-1.06) | 0.99 (0.91-1.10) |
| RHR | High | 0.94 (0.80-1.08) | 0.95 (0.69-1.22) | 0.82 (0.71-0.93) | 0.97 (0.82-1.12) | 0.82 (0.71-0.93) |
| PP | Low | 1.01 (0.88-1.13) | 1.16 (0.93-1.39) | **1.13 (1.03-1.23)** | 1.12 (0.99-1.26) | **1.13 (1.03-1.22)** |
| PP | High | 0.85 (0.72-0.98) | 0.71 (0.45-0.96) | 0.80 (0.70-0.90) | 0.85 (0.70-0.99) | 0.81 (0.71-0.91) |
| Physical fitness | Low | **1.42 (1.29-1.55)** | **1.90 (1.66-2.15)** | **1.25 (1.16-1.33)** | **1.45 (1.30-1.60)** | **1.22 (1.13-1.31)** |
| Physical fitness | High | 0.6 (0.32-0.87) | 0.27 (-0.38-0.92) | 0.77 (0.56-0.98) | 0.60 (0.32-0.87) | 0.78 (0.56-0.99) |
| BMI | Low | 1.19 (0.85-1.53) | 0.63 (-0.25-1.52) | 1.20 (1.03-1.38) | 0.92 (0.53-1.31) | 1.19 (1.02-1.37) |
| BMI | Overweight | **1.33 (1.20-1.46)** | **1.63 (1.40-1.86)** | 0.89 (0.74-1.04) | **1.37 (1.23-1.51)** | 0.90 (0.74-1.05) |
| BMI | Obese | **1.45 (1.22-1.69)** | **2.02 (1.61-2.42)** | **1.37 (1.09-1.64)** | **1.82 (1.58-2.07)** | **1.40 (1.12-1.67)** |

*Note.* Associations adjusted for birth year, sex, highest education of either parent, depression, anxiety, and SUD. Reference class was set to normative/median values. Bolded estimates are significant at α < .05.
